# Beam modeling and VMAT performance with the Agility 160‐leaf multileaf collimator

**DOI:** 10.1120/jacmp.v14i2.4136

**Published:** 2013-05-06

**Authors:** James L. Bedford, Michael D. R. Thomas, Gregory Smyth

**Affiliations:** ^1^ Physics Department The Institute of Cancer Research and The Royal Marsden NHS Foundation Trust Downs Road Sutton, Surrey SM2 5PT UK

**Keywords:** multileaf collimator, dose calculation, volumetric‐modulated arc therapy, treatment planning system

## Abstract

The Agility multileaf collimator (Elekta AB, Stockholm, Sweden) has 160 leaves of projected width 0.5 cm at the isocenter, with maximum leaf speed $3.5{\text{cms}}^{-1}$. These characteristics promise to facilitate fast and accurate delivery of radiotherapy, particularly volumetric‐modulated arc therapy (VMAT). The aim of this study is therefore to create a beam model for the ${\text{Pinnacle}}^{3}$ treatment planning system (Philips Radiation Oncology Systems, Fitchburg, WI), and to use this beam model to explore the performance of the Agility MLC in delivery of VMAT. A 6 MV beam model was created and verified by measuring doses under irregularly shaped fields. VMAT treatment plans for five typical head‐and‐neck patients were created using the beam model and delivered using both binned and continuously variable dose rate (CVDR). Results were compared with those for an MLCi unit without CVDR. The beam model has similar parameters to those of an MLCi model, with interleaf leakage of only 0.2%. The verification of irregular fields shows a mean agreement between measured and planned dose of 1.3% (planned dose higher). The Agility VMAT head‐and‐neck plans show equivalent plan quality and delivery accuracy to those for an MLCi unit, with 95% of verification measurements within 3% and 3 mm of planned dose. Mean delivery time is 133 s with the Agility head and CVDR, 171 s without CVDR, and 282 s with an MLCi unit. ${\text{Pinnacle}}^{3}$ has therefore been shown to model the Agility MLC accurately, and to provide accurate VMAT treatment plans which can be delivered significantly faster with Agility than with an MLCi.

PACS number: 87.55kd, 87.55km, 87.56bd, 87.56jk

## I. INTRODUCTION

The Agility multileaf collimator (Elekta AB, Stockholm, Sweden) has 160 leaves of projected width 0.5 cm at the isocenter.^(^ ^1^ ^)^ Maximum leaf speed is $3.5{\text{cms}}^{-1}$ and integrated dynamic leaf guides allow each whole leaf bank to further move by up to an additional $3.0{\text{cms}}^{-1}$. There are no backup collimators. Orthogonal to the direction of leaf motion, there are sculpted collimators which can move at up to $9.0{\text{cms}}^{-1}$. These characteristics promise to facilitate fast and accurate delivery of radiotherapy, particularly volumetric‐modulated arc therapy (VMAT).^(^ ^2^ ^)^

The basic radiation characteristics of the MLC have been evaluated previously.^(^ ^1^ ^)^ However, the presence of collimators in one direction in the beam's eye view and the absence in the other direction, the presence of the dynamic leaf guides, and the fast motion of the MLC leaves all create challenges for the treatment planning system in modeling the MLC. The first goal of this study is therefore to create a beam model for a commercial treatment planning system, ${\text{Pinnacle}}^{3}$ (Philips Radiation Oncology Systems, Fitchburg, WI), and to verify the accuracy of this beam model. The second goal is to use this beam model to evaluate the performance of the Agility MLC for delivery of VMAT. Head‐and‐neck cases are used for this performance evaluation, as these represent the most challenging cases in current clinical practice.^(^ ^3^ ^,^ ^4^ ^)^

## II. MATERIALS AND METHODS

### A. Agility and MLCi radiation heads

The Agility radiation head is considered in this study in comparison to the MLCi head (Elekta AB, Stockholm, Sweden), so the features of each are briefly reviewed. The 160 MLC leaves of the Agility head are supported by dynamic leaf guides which are not intended to be attenuating to radiation and which allow the leaves to move up to 15 cm over the central axis of the beam, with a speed of up to $6.5{\text{cms}}^{-1}$ (Table 1). Interdigitation is supported and there are no backup collimators moving in the direction of leaf motion. Moving in the orthogonal direction to the MLC leaves are specially shaped collimators which can travel over the central axis by 12 cm.

**Table 1:** Comparison of Agility and MLCi heads. Field sizes are defined at isocenter. In the ranges of travel, a negative value indicates the direction of insertion and a positive value indicates the direction of withdrawal.

*Characteristic*	*Agility Head*	*MLCi Head*
Maximum field size (cm)	40×40	40×40
Leaf pitch (cm)	0.5	1.0
X^a^ collimator range with respect to central axis (cm)	Not applicable	−12.5 to 20
Leaf guide range with respect to central axis (cm)	5 to 20	Not applicable
MLC leaf range with respect to guide (cm)	−20 to 0	Not applicable
MLC range with respect to central axis (cm)	−15 to 20	−12.5 to 20
Y^a^ collimator range with respect to central axis (cm)	−12 to 20	0 to 20
Focus – MLC distance (cm)	31.8	29.8
MLC thickness (cm)	9.0	7.5
Maximum MLC leaf speed (${\text{cms}}^{-1}$)	3.5	2.0
Maximum X^a^ collimator / leaf guide speed (${\text{cms}}^{-1}$)	3.0	2.0
Maximum Y^a^ collimator speed (${\text{cms}}^{-1}$)	9.0	1.5

a IEC61217 convention.

**Figure 1:**
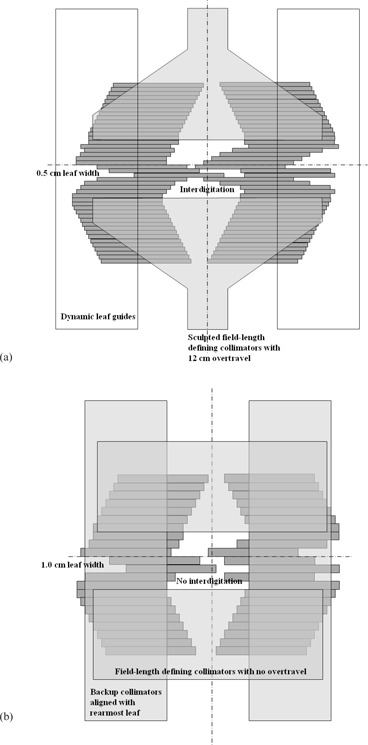
Comparative beam's eye views of (a) Agility and (b) MLCi multileaf collimators, showing the principal differences in design. This is a schematic representation only and is not to scale.

In contrast, the standard MLCi head has 80 leaves of 1 cm width at the isocenter, which can overtravel the central axis by 12.5 cm. There are backup collimators which follow the rearmost MLC leaves to minimize interleaf leakage, but these do not contribute to the leaf speed, like the dynamic leaf guides on the Agility head. The orthogonal field‐defining collimators are rectangular and they cannot travel over the central axis of the beam. Figure 1 illustrates these concepts. (Note that there is also an MLCi2 available which allows interdigitation; this is not considered in the present study.)

### B. Beam modeling

Depth doses and cross‐beam profiles were measured with a TW60008 p‐type Silicon diode detector (PTW, Freiburg, Germany) under a 6 MV Agility beam for square field sizes ranging from 3×3 cm to 40×40 cm in a plotting tank (PTW, Freiburg, Germany). Output factors were measured using a Type 2571 $0.6\;{\text{cm}}^{3}$ ionization chamber (Saint Gobain Crystals and Detectors, Reading, UK) in Solid Water (Radiation Measurements, Inc., Middleton, WI) at a depth of 10 cm. The focus‐to‐surface distance (FSD) was 90 cm for both the plotting tank measurements and the output factor measurements. A beam model was then created in ${\text{Pinnacle}}^{3}$ in accord with previously established procedures.^(^ ^5^ ^)^ Specifically, the automodeling function in ${\text{Pinnacle}}^{3}$ was used initially and then final adjustments were made manually, particularly for MLC and collimator transmission.

The combination of the dynamic leaf guides and the MLC leaves working together meant that the maximum leaf speed was $6.5{\text{cms}}^{-1}$, if all leaves in a bank were moving in the same direction, reducing to $3.5{\text{cms}}^{-1}$ for the case of some leaves travelling a given distance in one direction and other leaves travelling the same distance in the opposite direction. This behavior was not modeled explicitly in the treatment planning system, so the speed of the MLC leaves was constrained conservatively in ${\text{Pinnacle}}^{3}$ as $3\;{\text{cms}}^{-1}$. Furthermore, the minimum gap between opposing leaves was in principle 0.5 cm at the isocentric plane, so as to maintain a separation of 0.1 cm at the MLC itself, allowing for the rounded ends of the MLC (physical radius 17 cm). However, in this study, the minimum leaf separation (“dynamic leaf gap”) was set to 2 cm so as to ensure that the apertures were 2 cm or larger in width, thereby allowing the dose to be calculated with greater certainty. This was in accord with current clinical practice at this center.

Treatment fields and plans were transmitted directly to the accelerator control system by DICOM transfer. ${\text{Pinnacle}}^{3}$ defined two pairs of collimators, but one pair was redundant for the Agility head due to the absence of backup collimators. The Agility control system required this pair of collimators to be set to ±20 cm, so for the purposes of DICOM transfer, a simple script was executed after creation of treatment plans to set the collimator positions for the redundant collimators to ±20 cm. (This step is not necessary when using MOSAIQ (Elekta, Sunnyvale, CA) for treatment delivery.)

Nine specific fields were then created to test the beam model. These were a 5 cm×5 cm field on the central axis, a 10 cm×10 cm field on the central axis, a 30 cm×30 cm field on the central axis, a 4 cm×20 cm field on the central axis, a 20 cm×4 cm field on the central axis, a 20 cm×20 cm L‐shape field, a 20 cm×20 cm S‐shape field, a 5×5 cm field positioned 7.5 cm from the center of the field along one axis, and a 5×5 cm field positioned 7.5 cm from the center of the field along the other axis. Doses were calculated in a water phantom in ${\text{Pinnacle}}^{3}$. Depth doses and cross‐beam profiles were measured in a plotting tank for the L‐shape and S‐shape fields. For the L‐shape field, both central axis profiles and off‐axis profiles along the lines of the “L” shape were obtained. The same equipment was used as for measurement of the beam data (see above). A Type 2577 $0.2\;{\text{cm}}^{3}$ ionization chamber (Saint Gobain Crystals and Detectors) was then used to measure doses in various locations under all of these fields in Solid Water at 10 cm depth and 90 cm FSD to verify the accuracy of the model.

The performance of the beam model for dynamic fields was also briefly demonstrated. A 2 cm×20 cm field was swept dynamically from 10 cm to one side of the isocenter to 10 cm to the other side of the isocenter and the dose at the isocenter was measured using a Type 2571 $0.6\;{\text{cm}}^{3}$ ionization chamber. The gantry was at 0° for this test, the FSD was 90 cm, and the depth of the ionization chamber was 10 cm. The axis of the ionization chamber was oriented perpendicular to the direction of leaf motion. The dynamic field delivered 540 MU either in a smooth sweep or with static pauses every 2 cm, in which additional dose was delivered.^(^ ^2^ ^)^ The use of pauses created a situation in which a dynamic sweep and a series of matched static fields were superposed. In ${\text{Pinnacle}}^{3}$, the fields were modeled as a summation of apertures at intervals of 0.1 cm across the 20 cm leaf sweep.

### C. VMAT planning

VMAT treatment plans for five typical head‐and‐neck patients were created using the beam model. The clinical diagnoses for the five patients were: squamous cell carcinoma (SCC) of the base of the tongue, SCC hypopharnyx, SCC tonsil, follicular carcinoma of the thyroid gland, and metastatic ductal carcinoma of the breast. The clinical target volumes (CTVs) consisted of the region of primary disease (CTV1) and the bilateral neck nodes (CTV2). The planning target volumes were created by adding a uniform 0.3 cm margin to these CTVs, and the nodal PTV (PTV2) was excluded from the primary PTV (PTV1) so as to obviate conflicts in the optimization. Both PTVs were edited back from the skin surface by approximately 0.5 cm so as to prevent the treatment planning system from attempting to boost the dose in the build‐up region. The spinal cord and brainstem were contoured and planning risk volumes (PRVs) created by adding 0.3 cm margins. The whole parotid glands and the superficial parotid glands were also contoured. The prescribed dose was 65 Gy in 30 fractions to PTV1 and 54 Gy in 30 fractions to PTV2 simultaneously.^(^ ^6^ ^)^

The VMAT beam arrangement was a single anticlockwise arc from gantry 179° to gantry 181°. In the optimization, the principal objectives were to minimize the dose variation in the PTVs and to minimize mean dose to the parotid glands (aiming for below 24 Gy), while maintaining a maximum spinal cord PRV dose of 48 Gy to 1 ${\text{cm}}^{3}$ and a maximum brainstem PRV dose of 55 Gy to $1\;{\text{cm}}^{3}$. The maximum delivery time was specified as 400 s, and an additional constraint of 0.8 cm/° on MLC motion was applied, as for clinical head‐and‐neck VMAT protocols at this center. Without this constraint, on control points where the gantry was moving at considerably less than maximum speed thereby taking a relatively long time to pass through 1° of arc, the leaves would have been allowed to move a considerable distance in that time. The constraint prevented this from happening. ${\text{Pinnacle}}^{3}$ calculated the dose distribution according to the leaf positions at the discrete control points and by limiting the leaf motion, the actual leaf positions between the control points were constrained to be not far from those used for dose calculation, thereby improving accuracy. Note that with the gantry moving at full speed, the Agility MLC leaves can move 0.55 cm/° and the MLCi leaves can move 0.36 cm/°. If the gantry slows to around half maximum speed, the practical planning constraint of 0.8 cm/° starts to take effect.

The plans were compared with corresponding clinically treated plans for an accelerator with an MLCi head. In this case, the maximum leaf speed was set in the beam model to $2.0{\text{cms}}^{-1}$. The minimum leaf gap was 2 cm and the maximum delivery time was again 400 s.

The Agility and MLCi treatment plans were then delivered on their respective accelerators. The maximum dose rates of both Agility and MLCi accelerators were between 540 and 550 monitor units (MU) per minute. The plans on the MLCi unit were delivered using binned dose rate (i.e., 540 MU/min, 270 MU/min, 135 MU/min, etc.) as this was the only option available on this unit, but on the Agility unit the plans were delivered using both binned and continuously variable dose rate (CVDR) to investigate the effect of this factor on the resulting treatment time and delivery accuracy. The treatment plans were verified using a ${\text{Delta}}^{4}$ phantom (ScandiDos, Uppsala, Sweden)^(^ ^7^ ^)^ using a gamma evaluation for 3% of the dose to the primary PTV and 0.3 cm distance to agreement. Following the normal procedure at this center, measurements less than 20% of the dose to the primary PTV were excluded from the analysis so as to avoid considering irrelevant regions receiving low dose. However, for the large treatment volumes in this study, the 20% isodose encompassed almost the entire diode array, so that the number of diodes excluded were few. The results were considered in relation to a clinical pass criterion that ideally 95% of measurements, and an absolute minimum of 90% of measurements, should have a gamma value of less than unity. Since the ${\text{Delta}}^{4}$ phantom had been extensively commissioned and widely used at this center,^(^ ^7^ ^)^ confidence in the results was such that no further verification measurements were considered necessary for VMAT verification.

Statistical analysis was performed on the planning and delivery statistics using a Wilcoxon matched‐pair signed‐rank test in SPSS (IBM, Armonk, New York). Statistical significance was taken as p<0.05.

## III. RESULTS

### A. Beam modeling

**Figure 2:**
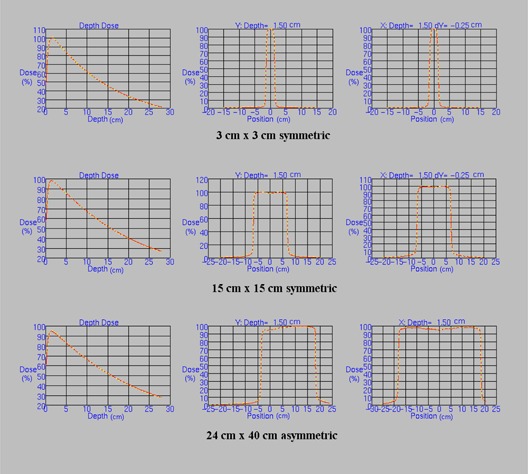
Comparisons of the Agility beam model (yellow dotted lines) with the corresponding measured data (solid red lines) for representative simple fields. These fields are among those used to produce the beam model. The central axis percentage depth dose and two orthogonal central axis profiles at 1.5 cm deep are shown in each case. The profiles in the direction of the multileaf collimator leaf motion are offset by 0.25 cm from the central axis so as to measure dose under a leaf rather than between leaves. Depth doses are normalized to 10 cm deep and profiles are normalized to the central axis. X and Y refer to IEC 61217, and positive X is directed towards X1 and positive Y is directed towards Y2. Distances are in cm.

The beam model has broadly similar parameters to those of an MLCi model, but with the MLC dimensions and transmission modified. The key parameters in the model are summarized in Table 2, and comparisons of the model with measured data are shown in Fig. 2.

**Table 2:** Summary of the ${\text{Pinnacle}}^{3}$ model parameters, with the parameters of the MLCi model for comparison.

*Parameter* ^a^	*Agility Value*	*MLCi Value*
Source A‐B dimension (cm)	0.01	0.01
Source G‐T dimension (cm)	0.01	0.01
Gaussian height (cm)	0.04	0.05
Gaussian width (cm)	1.0	1.0
Off‐axis softening factor	10.48	10.76
X^b^‐collimator transmission (%)	1.5^c^	9.5
Y^b^‐collimator transmission (%)	1.5	0.1
MLC transmission (%)	0.5	0.1
Leaf tip radius (cm)	17.0	15.0
Tongue‐and‐groove width (cm)	0.1	0.175
Interleaf leakage transmission (%)	0.2	3.0
Maximum gantry speed (°$s^{-1}$)	5.5	5.5
Maximum collimator speed (${\text{cms}}^{-1}$)	8.5	1.5
Maximum MLC leaf speed (${\text{cms}}^{-1}$)	3.0	2.0

a The reader is referred to Starkschall et al.(^5^) for a description of the parameters.

b IEC61217 convention.

c Not applicable in practice, as the Agility head does not have X‐collimators.

**Figure 3:**
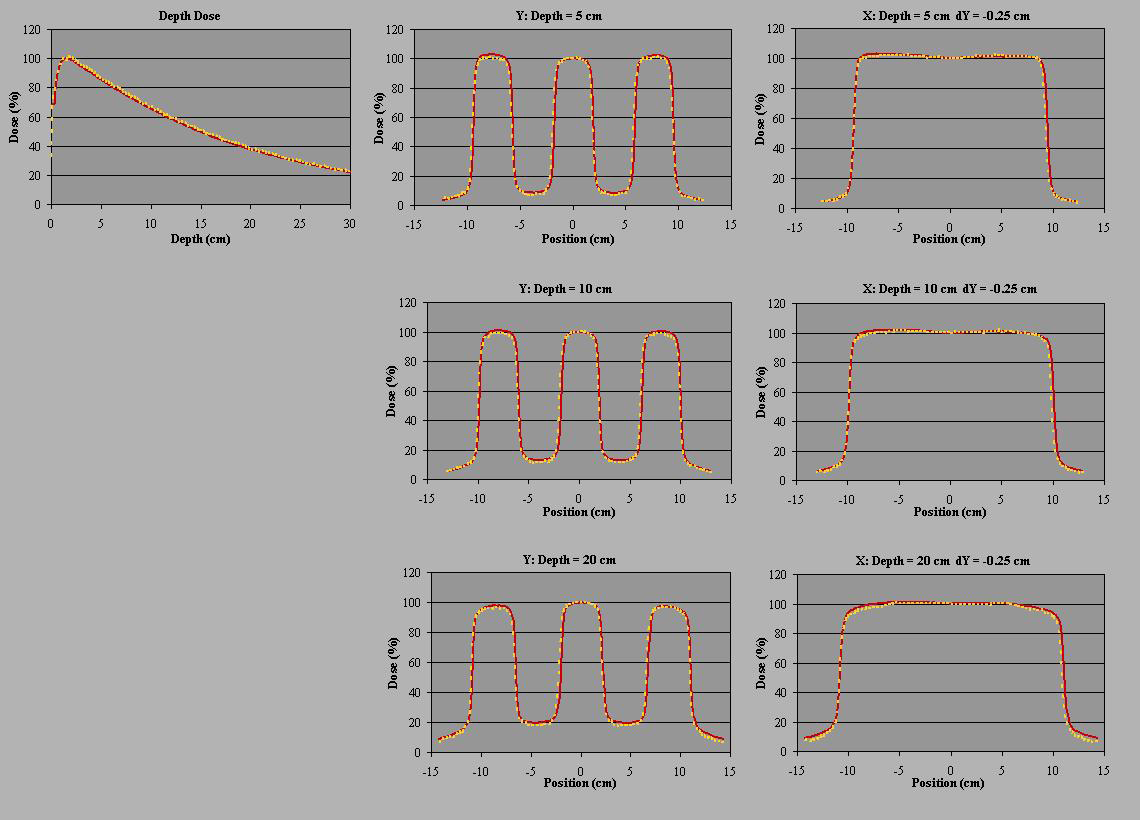
Comparisons of the Agility beam model (yellow dotted lines) with the corresponding measured data (solid red lines) for the S‐shape field. The central axis percentage depth dose and two orthogonal central axis profiles at 5 cm, 10 cm, and 20 cm deep are shown. The profiles in the direction of the multileaf collimator leaf motion are offset by 0.25 cm from the central axis so as to measure dose under a leaf rather than between leaves. Depth dose is normalized to 1.6 cm deep and profiles are normalized to the central axis. X and Y refer to IEC 61217, and positive X is directed towards X1 and positive Y is directed towards Y2. Distances are in cm.

**Figure 4:**
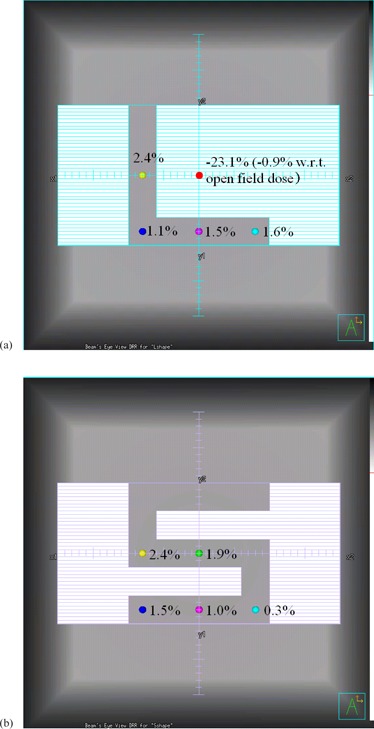
Typical verification results for the irregularly shaped fields at 10 cm depth in Solid Water. Planned doses are given with respect to measured doses: (a) L‐shape field, (b) S‐shape field.

The verification of irregular fields shows a good agreement between the measured and planned dose profiles for the L‐shape and S‐shape fields (Fig. 3). For the complete set of irregular fields, the mean agreement between measured and planned dose is 1.3% (planned dose higher), with a maximum difference of 3.2%. Figure 4 summarizes the absolute dose results for the main irregular fields.

The dynamic fields show an agreement between planned and measured dose of 0.5% (planned dose higher) without pauses and −1.1% (planned dose lower) with pauses.

### B. VMAT planning

**Figure 5:**
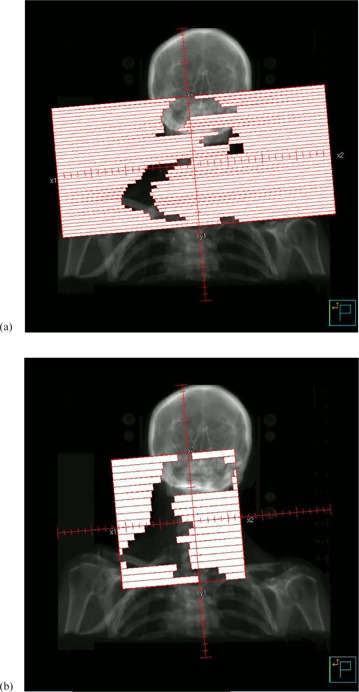
Beam's eye view of the control point at gantry angle 179° using (a) Agility and (b) MLCi.

**Figure 6:**
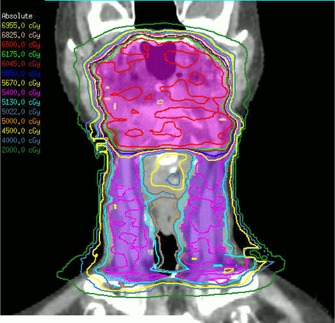
Representative coronal dose distribution for one of the Agility plans, showing the primary PTV (pink) and nodal PTV (purple).

The difference between Agility and MLCi collimation is illustrated in Fig. 5 for one of the VMAT head‐and‐neck plans, and a typical dose distribution created with the Agility model is shown in Fig. 6. Mean statistics are shown in Table 3 for the head‐and‐neck plans. The plans show comparable dose statistics to an MLCi unit, the monitor units being the only feature to show a statistically significant difference between the MLCi and the Agility.

**Table 3:** Mean statistics for the head‐and‐neck plans.

	*Constraint/Goal*		*Mean* Dose±1 *SD (Gy)* ^a^	
*Region Of Interest*	*Volume*	*Dose (Gy)*	*Agility*	*MLCi*
PTV1edited	99%	>58.5	61.7±0.4	61.6±0.6
	95%	>61.8	62.8±0.2	62.8±0.3
	50%	65	65.2±0.0	65.2±0.0
	5%	<68.3	67.1±0.2	67.1±0.3
	2%	<71.5	67.5±0.2	67.5±0.4
PTV2edited	99%	>48.6	51.2±0.4	51.4±0.3
	95%	>51.3	52.0±0.3	52.2±0.3
	50%	54	54.2±0.2	54.1±0.2
Spinal cord	Maximum	<46	44.3±1.0	44.3±1.1
Spinal cord	$1{\text{cm}}^{3}$	<45	40.8±1.2	41.0±0.4
Spinal cord PRV	$1{\text{cm}}^{3}$	<48	44.8±0.7	44.5±0.9
Brainstem	Maximum	<54	43.2±11.2	41.6±14.9
Brainstem	$1{\text{cm}}^{3}$	<50	35.9±15.0	35.6±15.4
Brainstem PRV	$1{\text{cm}}^{3}$	<55	42.5±10.4	41.6±12.9
Ipsilateral parotid	Mean	<24	36.5±6.1	37.3±6.7
Contralateral parotid	Mean	<24	30.3±5.5	31.0±5.2
Superficial parotids	Mean	<24	24.8±3.5	25.6±4.1
Total monitor units			531.9±66.4	477.1±27.3

a Items in bold are statistically significant at the p<0.05 level.

**Figure 7:**
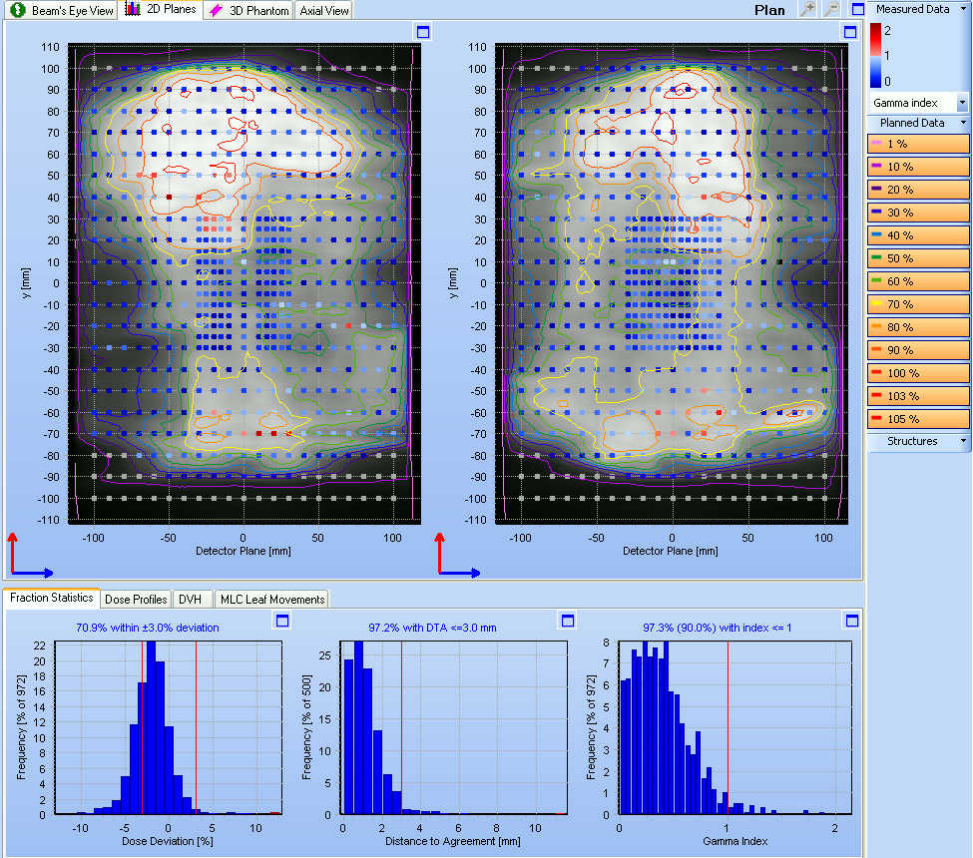
${\text{Delta}}^{4}$ results for an Agility delivery with CVDR. The upper panels show the two planes of diodes in the phantom. The grayscale distribution and isodoses show the planned dose, normalized to the primary PTV dose. The colored squares show the gamma value (3% and 3 mm) for the measured dose. The lower graphs are histograms of dose difference, distance to agreement, and gamma for the measured dose with respect to the planned dose. In this case, 97.3% of measurements have a gamma value of less than unity, the pass criterion of 90% being shown in brackets above the gamma histogram.

A typical verification result in ${\text{Delta}}^{4}$ for an Agility delivery with CVDR is shown in Fig. 7. Delivery times and verification results are summarized more fully in Table 4. Results are shown for both binned dose rate and CVDR, to facilitate a like‐for‐like comparison with the MLCi unit, for which binned dose rate has been used.

**Table 4:** Mean ± 1 SD statistics for beam delivery and verification using binned dose rate and continuously variable dose rate (CVDR).

	*Agility* ^a^		*MLCi*
	*Binned Dose Rate*	*CVDR*	*Binned Dose Rate*
Delivery time (s)	171.4±10.4	132.6±7.4	281.8±100.0
Verification gamma (3%/3 mm)	95.0±2.9	95.2±2.8	96.2±1.3

a Items in bold are statistically significant at *the p* < 0.05 level, compared against the MLCi.

## IV. DISCUSSION

The ${\text{Pinnacle}}^{3}$ beam model for the Agility head is similar in quality to previously described beam models with static delivery.^(^ ^5^ ^–^ ^8^ ^)^ The model represents the Agility MLC accurately, thereby providing VMAT treatment plans which reflect the narrow MLC leaves, low leaf transmission and fast leaf speed. The similarity of the beam model to that for an MLCi head is not surprising, as the accelerator itself is the same for the two heads and only the collimating device is different. Some of the ${\text{Pinnacle}}^{3}$ parameters are not necessarily physical, but provide dosimetric results which match the delivered dose. For example, the MLC transmission of the Agility head is not actually higher than that of the MLCi head. Note also that the tongue‐and‐groove width used in the model is not physically real, as the accelerator has defocused leaves without a physical tongue and groove.^(^ ^9^ ^,^ ^10^ ^)^ This approximated behavior in ${\text{Pinnacle}}^{3}$ and other treatment planning systems is well known.^(^ ^5^ ^)^ The physical speeds of the gantry, collimators, and MLC leaves are also chosen to be slightly conservative so as not to stress the delivery system, in accord with our clinical VMAT procedure. The delivery system adjusts the actual speeds according to its own accelerator‐specific variables.

The results of the irregular field tests show that the beam model predicts dose mostly to within 2%, with a maximum discrepancy of 3%. These results vary by ±1%, depending upon whether the Solid Water used for the practical measurements is modeled in ${\text{Pinnacle}}^{3}$ as a water phantom of unit density, a density‐overridden phantom of the exact density ($1.03{\text{gcm}}^{-3}$) of the Solid Water, or a volume represented by a CT scan, in conjunction with an appropriate CT‐to‐density table. However, the overall results in these cases are similar to those given in Fig. 4 for a water phantom. These results are very similar to those reported previously.^(^ ^8^ ^,^ ^11^ ^,^ ^12^ ^)^ The maximum discrepancy of 3% is unlikely to manifest itself clinically, but the general trend for planned dose to be around 1% higher than measured dose in irregular fields manifests itself in the VMAT verification results, where measured dose is typically 1%–2% low on average (see Fig. 7). The accuracy of the calculated dose in the irregular fields depends mostly on the accuracy of the calculated head scatter and phantom scatter, so that adjustment of the beam model itself to improve the results is ineffective. A predominance of long, narrow apertures in VMAT beams also tends to cause the overestimation of output factor, which similarly contributes to slightly low measured dose during VMAT verification. This is also difficult to adjust. Methods for improving the calculated output factors are the subject of ongoing work. Despite these limitations, the dynamic fields demonstrate that the beam model is capable of accurately predicting the dose delivered by a dynamic sliding window.

Narrower leaf width and lower transmission have both been shown to improve dose distributions for fixed‐gantry intensity‐modulated radiotherapy (IMRT).^(^ ^13^ ^,^ ^14^ ^)^ Although neither of these studies is based specifically on the Agility head, the studies indicate that improvements in target coverage, mean parotid dose, and dose conformality are possible with narrower leaf width and lower interleaf transmission. In this study, the plans for the Agility and MLCi units are roughly equivalent. There is an indication in the results that the parotid mean dose may be lower with the Agility head than with the MLCi head, but this is not statistically significant. However, this is not to say that the head does not provide superior dose quality in other situations. For example, treatment of brain tumors with conformal static beams or conformal arcs may benefit from the narrow leaves, due to the ability to closely follow the shape of the PTV in the beam's eye view. The situation may also be different with VMAT compared to static‐gantry IMRT. It is likely that in VMAT delivery, the motion of the leaves spreads out the distribution of dose so that the effect of the narrow leaves is less noticeable. The results of this study are, of course, also specific to the ${\text{Pinnacle}}^{3}$ treatment planning system, although comparable results are expected from other treatment planning systems.

The faster leaf speed of the Agility MLC is shown to be very beneficial for treatment delivery. The most prominent result in this study is that VMAT can be delivered extremely efficiently with the Agility head. Even with binned dose rate, which can be directly compared with delivery on the MLCi unit, there is a 40% reduction in delivery time and, with CVDR turned on, which is the normal delivery mode for the Agility accelerator, the delivery time is reduced by over 50%. The CVDR has been shown in other studies to improve the delivery speed of an MLCi unit by around 20%^(^ ^15^ ^,^ ^16^ ^)^ and this effect is also present with the Agility head. The overall result is that a high‐quality dose distribution is delivered in just over two minutes. The verification results show that the accuracy of delivery is not compromised by this speed. The gamma results meet the clinical criterion used at this center, which is normally met by VMAT head‐and‐neck plans delivered on an MLCi unit. Note that the verification accuracy is considerably in excess of the 85% of measurements within 5% and 5 mm required by ICRU 83.^(^ ^17^ ^)^

## V. CONCLUSIONS

${\text{Pinnacle}}^{3}$ models the Agility MLC accurately, thereby providing VMAT treatment plans which reflect the narrow MLC leaves, low leaf transmission, and fast leaf speed. These treatment plans are dosimetrically equivalent to those for an MLCi unit, and delivery of VMAT on the Agility unit is significantly faster than on the MLCi. Following the measurement of beam data, commissioning of the beam model, and verification of the dose distributions, the Agility head is fit for clinical use.

## ACKNOWLEDGMENTS

We are grateful to Elekta AB and Philips Radiation Oncology Systems for their collaborations in this project. We would also like to thank Mr. A. Warrington for his leadership throughout the study, and various other members of The Royal Marsden NHS Foundation Trust for their assistance with collecting beam data and for engineering support.
